# REAPER: a project-centric workflow layer for comparative repeatome analysis

**DOI:** 10.3389/fpls.2026.1855960

**Published:** 2026-08-31

**Authors:** Daniil S. Ulyanov, Anna I. Yurkina, Viktoria S. Voronezhskaya, Alana A. Ulyanova, Pavel Yu. Kroupin, Mikhail G. Divashuk

**Affiliations:** 1 All-Russia Research Institute of Agricultural Biotechnology, Moscow, Russia; 2 School of Biological and Medical Physics, Moscow Institute of Physics and Technology, Dolgoprudny, Moscow, Russia

**Keywords:** comparative genomics, repeatexplorer2, repeatome, reproducible workflows, satellite DNA, short-read sequencing, snakemake, TAREAN

## Abstract

**Introduction:**

Repeatome characterization from short-read sequencing data is widely performed using RepeatExplorer2/TAREAN. However, long-lived multisample projects and explicit comparative designs are often executed as *ad hoc* command sequences that are hard to version, rerun, and monitor on shared compute environments — a gap that motivates a project-centric workflow layer for repeatome analysis.

**Methods:**

We present REAPER (Repeatome Extended Analysis Pipeline—Execution and Reporting), a project-centric workflow layer that couples a modular Snakemake pipeline with a Python project manager to enforce a stable on-disk layout and configuration-driven execution for single-sample and comparative repeatome analyses. REAPER does not implement a new repeat-discovery algorithm; it is an orchestration layer, and biological accuracy for clustering and satellite calling depends on the underlying RepeatExplorer2/TAREAN and satMiner methods it coordinates. REAPER standardizes: Read QC Deterministic subsampling and preparation RepeatExplorer2/TAREAN execution via seqclust, with satMiner-inspired iterative assembly Post-TAREAN BLAST-based annotation against curated repeat collections (optionally including taxon-scoped NCBI-derived resources with freshness checks) Optional graph-based comparative reports The pipeline makes comparative read allocation, prefix policy, and analysis-ready tables explicit; caching supports incremental reruns and structured logs support monitoring. Performance was assessed using a *Triticeae* short-read dataset (five samples), with rule-level logging of runtime and memory across pipeline stages.

**Results:**

Rule-level performance logs show that graph-based clustering dominates runtime and memory, while QC and preparation steps are lightweight by comparison. Graph-report annotations for the Triticeae project additionally link high-ranking clusters to established repeat markers — including pTa794- and pSc119-class entries in curated databases.

**Discussion:**

These findings illustrate biologically interpretable outputs (recovery of known *Triticeae* repeat markers) alongside quantitative performance metrics (identification of graph-based clustering as the dominant computational cost). By making comparative read allocation, prefix policy, and analysis-ready tables explicit — and by supporting caching and structured logging — REAPER supports reproducible comparative repeatome analysis in evolving multisample projects. As an orchestration layer rather than a discovery algorithm, REAPER's contribution lies in reproducibility, monitorability, and comparative-analysis infrastructure, with biological accuracy remaining contingent on the underlying RepeatExplorer2/TAREAN and satMiner methods.

## 1 Introduction

High-throughput short-read sequencing and graph-based clustering algorithms have made it routine to characterize genome repeatomes and design cytogenetic probes directly from unassembled data. In plant and animal cytogenomics, pipelines such as RepeatExplorer2 with its TAREAN module, typically deployed as Galaxy workflows, have become central by enabling *de novo* identification, quantification, and visualization of transposable elements and satellite DNAs from low-coverage datasets (Novák et al., 2010, 2013, 2017, 2020). Complementary tools such as RepeatProfiler support comparative visualization and profiling of repetitive DNA from low-coverage short reads (Negm et al., 2021), while curated repeat databases [e.g., Repbase-like resources (Bao et al., 2015), Dfam (Storer et al., 2021), msRepDB (Liao et al., 2022), PGSB-REdat (Spannagl et al., 2017), TREPET (Kroupin et al., 2023)] provide reference catalogs of repeat families and satellite sequences. Other widely used approaches for repeatome characterization from unassembled short reads include dnaPipeTE, which assembles and quantifies repetitive elements from low-coverage genomic data (Goubert et al., 2015). For satellite DNA-focused mining from Illumina data, satMiner provides a toolkit of scripts and workflows used in “satellitome” studies and related surveys (Ruiz-Ruano, 2015, 2026). Recent satellitome-scale studies further demonstrate the value of standardized, repeatable pipelines for satellite discovery and comparative analysis across taxa (De Lima and Ruiz-Ruano, 2022). More recently, assembly- and long-read-aware pipelines complement short-read clustering approaches by enabling satellite discovery and structural characterization directly in complex genome assemblies and raw long-read datasets (Volarić et al., 2024; Kirov et al., 2022).

Taken together, these tools define a rich methodological ecosystem for repeatome analysis, but they are usually accessed either as web services or as loosely scripted command-line tools. This mode of use makes it difficult to maintain long-lived, multiproject datasets, to express systematic comparative designs, and to guarantee reproducible, configuration-driven workflows that can be versioned and rerun on different HPC environments. In many laboratories, repeatome analyses therefore remain a mixture of *ad hoc* scripts, manual Galaxy runs, and locally curated repeat collections. General-purpose workflow managers help with reproducibility, but they do not by themselves provide a domain-specific, project-centric representation of comparative repeatomics: persistent sample identity and naming across runs, consistent propagation of scientific parameters across multiple samples and comparisons, incremental reruns that touch only the affected parts of a long-lived project, and structured outputs that remain comparable as projects evolve. REAPER is designed to make these project-level concerns explicit and auditable while still executing established tools for clustering and annotation. REAPER is a workflow and project-management layer for running RepeatExplorer2/TAREAN and downstream annotation in a project-centric, configuration-driven way. It combines (i) a modular Snakemake workflow for read QC, deterministic subsampling, seqclust execution, and post-processing; and (ii) a Python project manager that enforces a stable on-disk layout and registers samples and comparative designs. Scientific parameters (read budgets, TAREAN thresholds, satMiner depth of iterations, BLAST cutoffs, and reference sets) are stored in YAML and applied consistently across runs. REAPER focuses on operational concerns that recur in repeatome projects: representing projects and comparisons explicitly, separating scientific settings from command lines, and rerunning only the parts of a workflow that change when inputs or parameters are updated. In practice, this is implemented using Snakemake’s DAG execution, per-rule logging, and a small set of validation and monitoring utilities to support long-running multisample runs. In this work, we contribute the following:

1. A project-centric representation and CLI for repeatome datasets and comparative designs.
2. A modular Snakemake workflow that standardizes deterministic preparation, RepeatExplorer2/TAREAN execution, and satMiner post-processing.
3. Operational artifacts (logs, structured outputs) that support monitoring and incremental reruns.
4. A graph-based reporting layer that summarizes cluster relationships and components for comparative interpretation.
5. Optional oligo/probe BLAST support to evaluate candidate probe sequences against curated or project-built repeat databases.

Together, these items consolidate project-centric management, reproducible Snakemake execution, operational monitoring, and comparative reporting in one layer.

## 2 Materials and methods

### 2.1 Overall architecture

**Figure 1:**
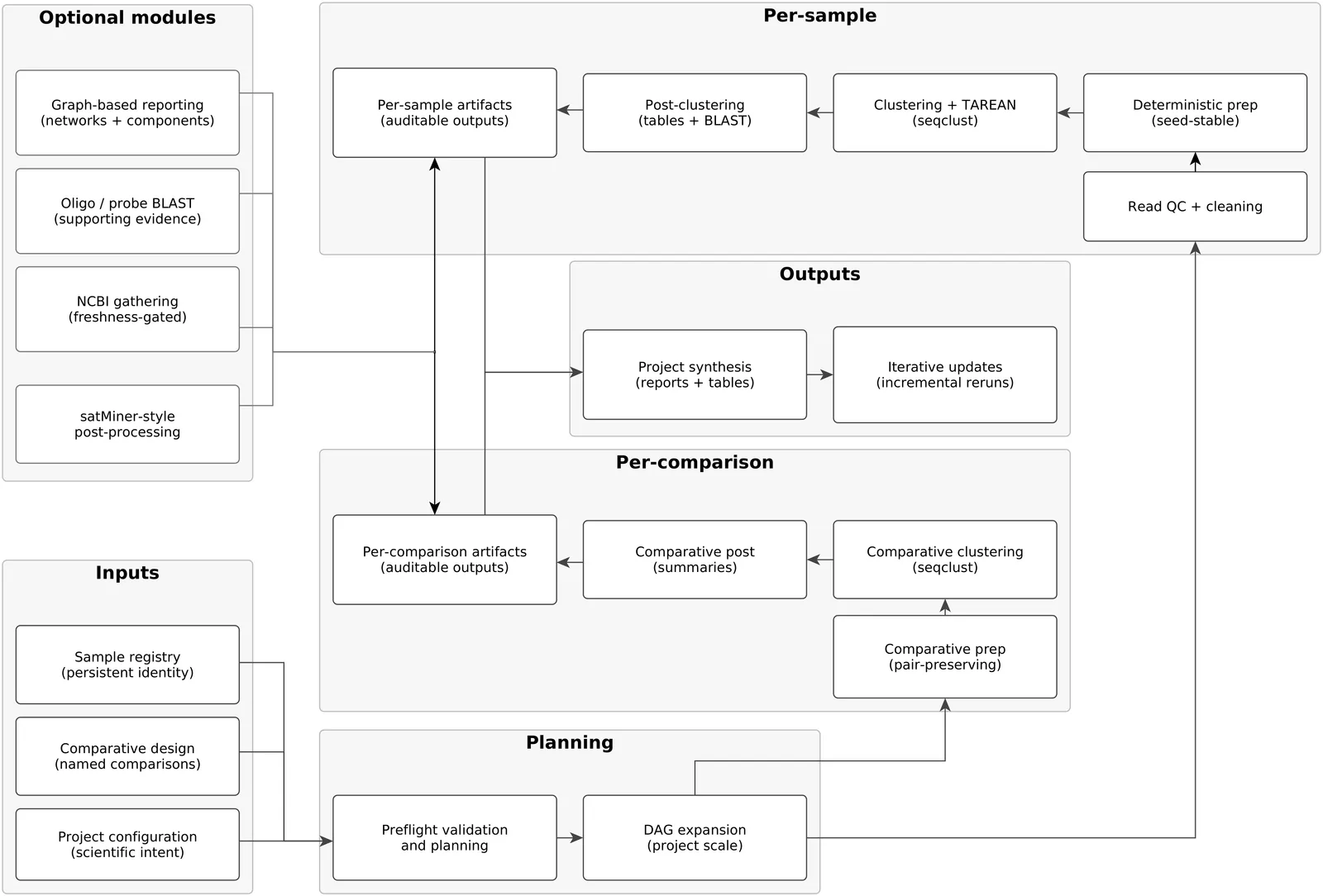
System-level architecture of REAPER. Inputs (YAML project configuration, a persistent sample registry, and declarative comparative analyses) are validated in a preflight step and expanded into a Snakemake DAG. The DAG executes two main branches: a per-sample pipeline and a per-comparison pipeline. Optional modules (taxon-scoped NCBI reference gathering, satMiner-inspired post-processing, graph-based reporting, and oligo/probe BLAST support) feed into the artifact layers. Final outputs are aggregated into project-level synthesis reports and support incremental reruns as projects evolve.

REAPER is implemented as a modular Snakemake workflow that orchestrates standard repeatome tools in a reproducible, project-aware fashion (Köster and Rahmann, 2018). The workflow codebase is organized into modular rule components for core analysis, reporting, and configuration validation, while orchestration logic and post-processing are implemented in Python 3 (**Figure 1**). Readers seeking full replication detail beyond the summary given here should consult the repository’s PROJECT_MANAGER.md, README.md, and INSTALLATION.md, which document project layout, environment setup, and CLI usage in full.

Scientific configuration is stored as YAML (projects/global_config.yaml). In the evaluated deployment, the workflow uses three pinned Conda environments (core QC/BLAST/post-TAREAN; RepeatExplorer2/TAREAN via seqclust; R-based graph reporting) rather than containerized execution, which isolates stacks at the cost of extra environment management. External tools include BBDuk/BBMap and FastQC for read quality control (Bushnell et al., 2017; Babraham Bioinformatics, 2010), RepeatExplorer2/TAREAN for graph-based clustering and satellite detection (Novák et al., 2010, 2013, 2017, 2020), integrated satMiner iterative assembly approach (Ruiz-Ruano, 2015, 2026), and NCBI BLAST+ for sequence annotation and database operations (Camacho et al., 2009). For reproducibility, the evaluated deployment uses pinned Conda environments referenced by lockfiles under envs/locks/(reportr, repeatexplorer, reportr_graph).

### 2.2 Project representation and configuration

Projects are represented as first-class entities under a common project/directory, each with machine-readable metadata (project_metadata.yaml) and a standardized on-disk layout. Within each project, samples are organized into subdirectories containing symbolic links to raw reads (raw_reads/), cleaned reads (filtered_reads/), assemblies, TAREAN outputs, and FastQC reports. A single global configuration file (projects/global_config.yaml) holds both global defaults (thread counts, memory limits, cache directories, temporary storage, deterministic PYTHONHASHSEED, target reads per assembly) and project-specific parameters (taxonomy, curated NCBI repeat sets, TAREAN and assembly settings, per-sample genome sizes, and FASTQ locations). Comparative analyses are defined declaratively as named sets of samples together with their intended comparisons, including genome-size-aware proportions that govern how reads are allocated to joint assemblies and comparative BLAST runs. These proportions are derived directly from the per-sample genome_size estimates recorded in the project configuration as described in the project’s README.md, so joint assemblies for taxa of markedly different ploidy or genome size receive read allocations scaled to their relative genome sizes rather than a flat per-sample split. In the current implementation, this scaling is applied without an automated check on the resulting read-depth ratio; REAPER does not yet warn when a large genome-size disparity risks under-sampling low-abundance satellites in the larger genome (see *Discussion*). A dedicated Python command-line interface (project_manager.py) automates creation of new projects, registration of samples with enforced metadata, assignment of deterministic unique per-sample read prefixes (by default the uppercased sample identifier, with a hash-based suffix only if another sample already uses that prefix), and structural and configuration validation of projects before execution. This scheme keeps labels stable in prepared FASTA; in comparative runs, seqclust -P is set to at least the longest prefix in the comparison (see Implementation details) so short prefixes do not collide across samples. A schematic overview of project layout and configuration is provided in PROJECT_MANAGER.md and related repository documentation. A minimal example of the global configuration intent is shown in **Supplementary File S1** (the repository configuration contains additional validated fields and defaults).

These configuration patterns express scientific choices (read budgets, clustering thresholds, taxonomic scope) declaratively, enabling version control and incremental reruns across projects. In practice, several additional configuration-driven features materially affect both biological scope and operational cost and are therefore worth highlighting explicitly in the manuscript:

1. Iterative assembly depth and masking policy: satMiner-inspired “RepeatExplorer → build reference from cluster contigs → filter unmapped reads → rerun” loops are controlled by global.iterative_assembly and per-project overrides (e.g., depth, mode), with deconseq.* fields governing strictness (removeifeitherbad) and reference size (iterative_reference_coverage_fraction).
2. Freshness-gated taxon repeat resources: when global.ncbi_gathering.enabled is true, NCBI-derived project FASTA assets can be checked against max_age_days before comparative annotation to keep “moving targets” explicit and auditable.
3. Incremental post-processing reuse: global.incremental_processing enables reusing previously computed BLAST result tables when inputs/parameters are unchanged, reducing rerun cost for iterative manuscript-era projects.
4. Graph-report scalability knobs: global.post_tarean_params.graph_report includes safety caps (e.g., maximum nodes/edges for interactive rendering) and permissive/default BLAST edge filters, making report complexity tunable without changing code.

### 2.3 Workflow overview

The workflow itself is decomposed into a series of well-delimited steps that together form a Snakemake DAG:

1. Read quality control and cleaning—raw paired-end Illumina reads undergo quality control and filtering using FastQC and BBDuk with parameters drawn from YAML configuration; cleaned reads are written to a dedicated filtered-read location and are marked with completion tokens to support incremental execution.
2. Deterministic subsampling and preparation for clustering—reads are deterministically subsampled to configurable per-sample read budgets using one configured seed value (pythonhashseed) propagated through both sampleseed and PYTHONHASHSEED in canonical workflow paths. Inputs are then converted into RepeatExplorer-compatible FASTA files while preserving stable per-sample prefixes.
3. Graph-based clustering (RepeatExplorer2/TAREAN)—clustering is executed via the RepeatExplorer2/TAREAN stack using the upstream seqclust entry point built in a local repex_tarean/checkout; per-project configuration drives parameters including mincl passed directly to seqclust -m as a proportion, thread limits under runtime CPU capping, and effective seqclust memory (-r, kB) resolved from node RAM policy. The pipeline coordinates isolated Rserve ports so multiple samples can be run concurrently on shared infrastructure.
4. Post-TAREAN consolidation and annotation—clustering consensus sequences and cluster metrics are consolidated into structured tables. For comparative analyses, repeat FASTA inputs are constructed across samples, and consensus sequences are annotated using NCBI BLAST+ against curated taxon-specific repeat collections (with optional probe/oligo evidence).
5. Reporting and comparative summaries—downstream steps assemble satMiner-style rich tables and derived summaries (e.g., divergence/abundance views and optional RepeatMasker profiling) and can additionally generate interactive graph-based reports that summarize repeat similarity relationships for comparative interpretation.

RepeatExplorer2 is invoked from a local repex_tarean/checkout populated by the repository installer (install_reportr.py; README.md). Key parameters used in the evaluated configuration are stored in projects/global_config.yaml and applied consistently across samples and comparisons. In brief, read cleaning uses BBDuk with bbduk_params: ktrim=r k=20 mink=10 hdist=2 threads=10 and an explicit adapter FASTA (read_cleaning.adapters: adapters/adapters.fa), with FastQC run using fastqc_threads: 10. Read preparation deterministically subsamples random_n: 8,000 read pairs by propagating global.pythonhashseed into the preparation stage. Clustering parameters are resolved from tarean_params (e.g., paired: true, assembly_min: 2, mincl: 0.0001, options: ILLUMINA_SENSITIVE_BLASTPLUS, domain_search: DIAMOND), with comparative prefix safety controlled by tarean_params.prefix_length and the comparison-specific maximum prefix length. Cluster granularity for closely related repeats—including homoeologous, subgenome-specific satellites differing by only a few SNPs—is governed by this same mincl threshold (passed to seqclust -m as a proportion of the read set); REAPER does not currently expose a dedicated SNP-aware or subgenome-specific stringency parameter beyond mincl. Comparative BLAST filtering uses comparative_params defaults (blast_evalue: 1e−5, min_identity: 80, min_coverage: 80), while graph-report edge construction uses a permissive default filter in post_tarean_params.graph_report (tasks megablast and dc-megablast, coverage types near-full and composite). For satellite DNA-focused downstream analysis, REAPER includes a satMiner-inspired post-processing module that operates on RepeatExplorer2/TAREAN outputs. This module reproduces key satMiner-style steps (e.g., consensus collection across iterations, divergence/abundance summaries, optional RepeatMasker-based profiling, and summary reporting) within the Snakemake execution model, enabling the same project-centric provenance, logging, and incremental reruns used for the core workflow. Attribution and scope: (i) REAPER-native components include Snakemake rules, YAML-driven orchestration, the project manager, graph-report and BLAST consolidation glue, and validation utilities. (ii) Algorithmic and reporting patterns adapted from satMiner (Ruiz-Ruano, 2015, 2026) are reimplemented or wrapped in that layer. For downstream interpretation and probe-design workflows, REAPER can additionally generate graph-based cluster relationship reports (connected components and interactive network visualizations) and can run oligo/probe query sets through BLAST against curated or project-built databases derived from the project’s consensus tables. In the current implementation, graph reporting builds similarity edges by BLASTing query consensus sequences against a project’s local database constructed from rich consensus tables, applies cached edge construction to avoid repeating identical searches, clusters the resulting similarity graph into connected components, and renders an interactive HTML report using visnetwork. Annotation of cluster-consensus sequences uses a tagged-consensus strategy (singular and tripled repeat monomer query variants, with a task ladder) against a project NCBI repeats database and incorporates an oligo/probe database to carry probe evidence into the reported nodes.

### 2.4 Implementation details relevant to reproducibility

REAPER makes several operational choices explicit because they affect reproducibility, performance, and interpretation of results:

1. Three-environment execution model: core QC/BLAST/post-processing run in the reportr environment; RepeatExplorer2/TAREAN executes in the repeatexplorer environment using repex_tarean/seqclust; and interactive graph report rendering runs in reportr_graph. This keeps the clustering and visualization stacks isolated from the main workflow environment.
2. Configuration hierarchy and resource policy: scientific parameters are resolved from projects/global_config.yaml using a strict hierarchy (global defaults under global → per-project overrides under projects → rule-specific handling in Snakemake). For clustering, the project-level tarean_params.threads and tarean_params.memory_limit can be expressed as either absolute values or percentages; in the evaluated *Triticeae* configuration, both are set to 80% (e.g., threads: 28, memory_limit: 80%, r_value: 80%). At runtime, REAPER detects node CPU/RAM and converts percentage values to effective seqclust limits by applying the configured fraction and then clamping to the Snakemake execution budget (e.g., --cores) and any project overrides. This “use most of the node but remain schedulable” policy is configurable per deployment and is intended to produce stable, comparable performance logs while preventing accidental oversubscription on shared nodes.
3. Unified parameter resolution across solo and comparative runs: solo and comparative clustering resolve parameters using the same logic and produce consistent logs and outputs, enabling comparable performance summaries.
4. Comparative read sampling in pairs: in comparative analyses, sampling is performed in read pairs (forward/reverse mates) to preserve pairing and prefixes; pair allocations are proportional to the configured genome sizes.
5. Comparative prefix safety (-P): comparative seqclust resolves -P as the maximum of configured minimum prefix length and the longest sample prefix in the selected comparison, reducing sample-identity ambiguity from prefix collisions.
6. NCBI freshness gate for comparative post-processing: when NCBI gathering is enabled, comparative post-TAREAN analysis is gated by project freshness checks (check_ncbi_freshness and ensure_ncbi_fresh_for_project) evaluated against max_age_days.
7. Deterministic seed propagation in canonical paths: the current canonical prep paths preserve the configured seed value across solo and comparative runs.
8. Centralized logging path: workflow and rule logs resolve through global.log_dir (LOG_DIR) across core, comparative, NCBI, validation, and progress-tracking modules.
9. *Cleanup and output semantics*: an optional cleanup mode removes heavy intermediate databases, so reported outputs are defined in terms of lightweight stable files (e.g., CLUSTER_TABLE.csv) and completion markers rather than large database artifacts.
10. Run provenance signatures: each clustering run records a parameter-resolution provenance signature at start and promotes it on completion, capturing the resolved clustering settings and the read-preparation configuration used to generate the prepared FASTA input.

Where NCBI repeat gathering is enabled, REAPER treats the taxon-scoped NCBI-derived FASTA as a project artifact: it can be gated by freshness checks, and BLAST databases are (re)built with parsable identifiers (e.g., -parse_seqids) to support downstream enrichment of reported accessions and titles.

### 2.5 Caching, validation, and monitoring

A central design feature of REAPER is explicit, parameter-aware caching and support for iterative workflows. The pipeline uses Snakemake’s native caching mechanism, but enforces a strict cache-key discipline in which hashes of input files are combined with the relevant configuration subset for each heavy step (read cleaning, subsampling and assembly, TAREAN clustering, BLAST database construction, BLAST annotation). Any change to underlying FASTQ content or to semantically relevant parameters (e.g. BBDuk filters, target read counts, TAREAN thresholds, BLAST cutoffs, composition of NCBI repeat sets) automatically invalidates only the affected cache entries. For post-TAREAN BLAST, when global.incremental_processing is enabled and a prior {subject}_blast_results.csv exists, the workflow can reuse that file instead of recomputing BLAST; otherwise, BLAST runs and persists the CSV for later reuse. Because caching is implemented at the level of physical files and Snakemake rules, it works seamlessly across multiple job submissions, cluster schedulers, and long-lived projects, while allowing users to add samples or refine parameters without rerunning unchanged parts of the workflow.

REAPER also supports observability for long-running clustering jobs through structured progress summaries derived from tool output, alongside a unified monitoring interface that can present progress views and stability-oriented wrappers for repeated execution on shared compute infrastructure.

REAPER also includes a validation, testing, and monitoring layer designed for long-term, multiproject use. A configuration validation component checks the coherence of scientific parameters and the internal consistency of the YAML configuration and verifies that referenced inputs and resources exist and are readable. Certain checks are intentionally non-fatal and emit warnings rather than aborting the workflow, reflecting experience from real deployments where overly strict validation can produce false positives. Dedicated workflow tests using synthetic projects ensure that core features such as project creation, comparative analysis wiring, BLAST coverage computation, and caching behavior remain stable across code changes. Monitoring utilities parse per-rule logs, track the progress and resource usage of assemblies and clustering jobs, and provide optional progress-tracking views for multiproject environments.

In addition to input existence checks, REAPER’s validation layer is designed to support reproducible, long-lived projects by reporting configuration completeness, tool availability, and warning-level checks for legacy patterns or suspicious hardcoding. These short validation reports are intended to be archived with run outputs to support troubleshooting and to document the execution environment at the time of analysis.

Deployment defaults follow host-side Conda as described in INSTALLATION.md. Optional Singularity/Apptainer or other OCI-style packaging may simplify multi-user HPC adoption in future releases but is not required for the evaluated configuration.

## 3 Results

REAPER has been exercised on internal and publicly derived projects, including *Triticeae* datasets for which we collected rule-level performance logs (wall-clock time, memory use, and CPU load). Below, we summarize the operational evidence available from these runs. A small illustrative public dataset (samples KA1–KA5) is additionally available at BioProject PRJNA1494421 to allow independent reproduction of the workflow end-to-end (see *Data availability statement*).

### 3.1 Biological plausibility (*Triticeae* graph report)

For the triticeae_F21FTSEUHT1241 project, the repeat-interaction graph post-processing produces cluster_annotations.tsv, in which high-ranking connected components link *de novo* consensus sequences to entries in the curated repeat/oligo database—for example, clusters annotated to pTa794-class and pSc119.2/AJ517288-related sequences widely used as *Triticeae* cytogenetic markers. This does not substitute for independent experimental validation, but shows that REAPER’s annotation layer recapitulates expected major repeat families rather than only operational metrics. For example, the short-unit consensus CL2_TR_1_x_118nt corresponds most closely to pTa794-class sequences, consistent with its short monomer length and high genomic abundance, while the longer-unit consensus CL125_TR_1_x_376nt groups with pSc119.2/AJ517288-related sequences, a repeat family commonly used for chromosome-arm identification in wheat and its wild relatives. These correspondences illustrate how REAPER’s annotation layer can anchor *de novo* consensus clusters to previously characterized cytogenetic markers, supporting downstream probe design and comparative interpretation.

A comparative run yields (i) a joint, pairing-preserving read preparation step for the selected sample set, (ii) a comparative clustering/TAREAN execution producing ranked consensus sequences, and (iii) post-processing outputs that summarize abundance, divergence, and homology/annotation evidence in structured tables and reports. For example, rank-1 consensus sequences from this comparative run include repeats such as CL2_TR_1_x_118nt and higher-length consensus units such as CL125_TR_1_x_376nt, consistent with a mixture of short and longer satellite candidates in the output catalog.

This case provides an illustrative operational cost snapshot rather than a comprehensive comparative survey: in the available artifacts for this comparison, joint read preparation completed in ~41–174 s, whereas the comparative clustering step required ~48–50 min and peaked at ~10–12 GiB peak RSS (resident set size), consistent with clustering dominating runtime and memory. Comparative end-to-end runs (through graph reports) are therefore presented as illustrative; solo-sample rule-level statistics below are the primary quantitative baseline.

### 3.2 Datasets and workloads

Projects and datasets analyzed: The evaluated runs include a *Triticeae* short-read evaluation case study (triticeae_F21FTSEUHT1241; 29 samples). Read budgets for *Triticeae* varied across samples (median 100,000 read pairs in the current configuration).

Toolchain and environments: The evaluated deployment uses the three pinned Conda environments summarized in **Supplementary Table 2**. Performance results reported here reflect execution on a shared-memory Linux HPC node with ~28 threads and ~180 GB RAM under the workflow’s resource policy.

### 3.3 Performance, scalability, and caching

Rule-level Snakemake benchmarks for triticeae_F21FTSEUHT1241 were generated from five independent repeated runs of five published accessions (as specified in projects/global_config.yaml; 50,000 reads per sample), for a total of 25 runs. Metrics are reported per run. The md5 file checksums of the outputs from the different runs matched. We take identical outputs combined with narrow timing spread as direct evidence of pipeline determinism. In the currently available benchmark artifacts, median (IQR) wall-clock times were 573 s (539–585 s) for clean_reads, 15 s (15–23 s) for prepare_reads, and 52 min (47–59 min) for run_tarean. Median (IQR) peak RSS values were ~4.41 GiB (4.25–5.84 GiB) for clean_reads, ~0.32 GiB (0.31–0.33 GiB) for prepare_reads, and ~10.23 GiB (7.28–11.26 GiB) for run_tarean (**Table 1**). Comparative read preparation remains I/O-heavy in the current artifacts (median ~174 s with multi-GiB peak RSS under the configured policy).

**Table 1:** Rule-level benchmarks for triticeae_F21FTSEUHT1241 (median and IQR; wall-clock in seconds; peak RSS in GiB).

Rule	Median wall-clock (s)	IQR low–high (s)	Median peak RSS (GiB)	IQR low–high (GiB)
clean_reads	573	539–585	4.41	4.25–5.84
prepare_reads	15	15–23	0.32	0.31–0.33
run_tarean	3,120 (~52 min)	2,820–3,540	10.23	7.28–11.26

Benchmark provenance and figure generation: per-rule runtime and resource values are taken from Snakemake log TSVs written under benchmarks/(one file per job, standard Snakemake performance logs format). Manuscript summary tables are generated deterministically from these TSVs by dashboard/build_benchmark_tables.py.

Beyond the single-sample performance logs summarized here, REAPER emits analogous rule-level performance logs for comparative analyses and for iterative runs where multiple clustering iterations are executed per sample. Repeated runs of comparative assemblies show the same deterministic behavior with matching checksums of the outputs. This broader performance logs coverage enables evidence-based planning for incremental project updates (e.g., adding samples, adding comparisons, or increasing iteration depth) without conflating those scenarios with the baseline single-run workload.

For run_tarean, REAPER derives an upper bound for seqclust threads and memory at runtime as 80% of detected node CPU and RAM, further capped by the Snakemake --cores budget and any project-level overrides. This makes performance logs comparable within an execution environment while reflecting the intended “use most of the node but remain schedulable” policy for long-running clustering steps.

REAPER’s incremental execution behavior is observable in routine Snakemake run logs: full (re)initialization executes many jobs when code or parameters change, whereas targeted updates execute only the minimal subset of affected jobs. This operational evidence aligns with the workflow’s caching/signature discipline: unchanged steps are not recomputed, and only the affected sample or comparison triggers downstream work.

### 3.4 Example outputs

REAPER produces a set of structured summary tables and reports intended for downstream interpretation and reuse. These include per-project summaries of satellite abundance and confidence classes, BLAST-based annotation tables for consensus sequences, and cache and runtime reports for monitoring pipeline performance. In the evaluated configuration, core outputs are written as CSV/TSV files suitable for downstream scripting, while the post-TAREAN modules can additionally render selected summaries as XLSX spreadsheets and DOCX/HTML reports when the corresponding Python libraries are present.

For a *Triticeae* sample, the satMiner-style post-TAREAN layer produces a “rich table” summarizing the most abundant clusters/consensus sequences, together with supporting summaries such as BLAST best-hit tables (including ×3 query variants), RepeatMasker-derived read-vs-consensus profiles (when enabled), and per-sample plots used for interpretability (e.g., abundance/divergence views).

**Figure 2:**
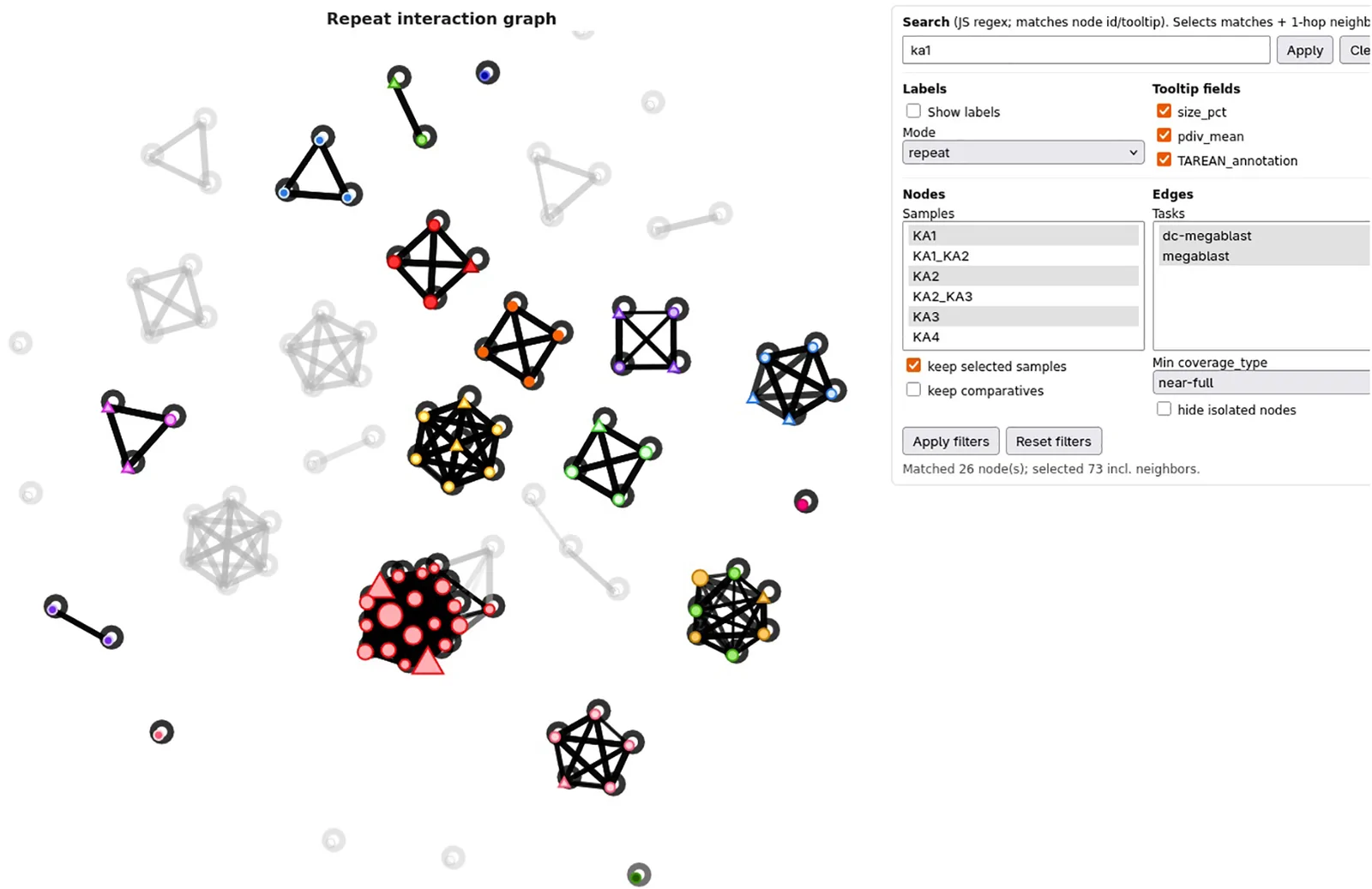
Repeat interaction graph report. An interactive similarity network built from TAREAN consensus sequences produced across per-sample and comparative analyses. Nodes are consensus repeats (identified by sample/comparison, iteration, and rank) and are annotated with best BLAST hits and optional oligo/probe matches. Edges connect repeats with significant local-database BLAST similarity (coverage types such as near-full/composite; tasks megablast/dc-megablast). The rendered interactive figure is the project’s reports/repeat_interaction_graph/graph.html, with connected components listed via cluster_sizes.tsv/clusters.tsv. Representative examples discussed in the text include the CL2_TR_1_x_118nt consensus (pTa794-class) and the CL125_TR_1_x_376nt consensus (pSc119.2/AJ517288-related), illustrating short- and long-unit satellite classes recovered by the pipeline.

At the project level, REAPER can additionally emit an interactive repeat-interaction graph report: a web-viewable network visualization accompanied by node/edge tables and connected-component summaries. Nodes are enriched with consensus-level annotation evidence (e.g., best-hit summaries and optional probe/oligo matches) so that cluster relationships can be interpreted alongside sequence annotation (**Figure 2**).

As a concrete example of the reporting layout, integration tests under tests/exercise small synthetic project configurations; full-structure illustrations—including TAREAN annotation columns, BLAST summaries, and top-cluster style tables—are produced by real runs such as the bundled *Triticeae* project outputs under projects/triticeae_F21FTSEUHT1241/.

To illustrate the contents of the top-cluster view, the tarean_annotation column [e.g., “Putative satellites (high confidence)”] is linked to an element cluster ID, its size (percent genome), and the best BLAST hit and coverage category.

Confidence outputs similarly allow to simultaneously estimate novelty and validity of results: extremely low-abundance clusters that lack usable TAREAN/BLAST evidence can be automatically flagged as DISCARD under configured cutoffs.

## 4 Discussion

REAPER is a workflow layer around established repeatome tools (RepeatExplorer2/TAREAN and curated repeat databases) for multiproject and comparative use. It standardizes project layout and parameter handling and exposes performance logs for operational monitoring (**Table 2**).

**Table 2:** Comparison of typical Galaxy-based RepeatExplorer2 usage and the REAPER workflow approach.

Aspect	Galaxy RepeatExplorer2 (typical)	REAPER
Parameter and sample wiring	History/UI-centric; manual tracking	YAML + project manager; version-controlled config
Multisample/comparative design	*Ad hoc* per session	Declarative projects and named comparisons
Reruns after input/param change	Often full manual redrive	Snakemake DAG + cache/signature boundaries
Operational artifacts	Variable	Rule logs, benchmarks, validation summaries by default

For comparative read pooling, proportional allocation depends on user-supplied genome size estimates; we recommend anchoring these in independent data (e.g., Plant DNA C-values and related compilations, flow cytometry, or k-mer-based surveys where assembly is unavailable) and revisiting estimates when taxonomic scope changes.

Sensitivity of cluster calling and abundance summaries to mincl (seqclust -m) is not exhaustively explored here; a small grid of values per genome class is a natural supplementary analysis for future work. This also bears on resolving closely related, homoeologous or subgenome-specific satellites: REAPER currently relies on this same mincl threshold for that purpose and does not expose a dedicated SNP-aware stringency parameter, so distinguishing near-identical subgenome variants may require manual tuning of mincl per comparison.

In day-to-day use, projects are defined in YAML, samples are registered once, and comparative analyses are expressed explicitly; subsequent parameter adjustments can be applied consistently across samples and projects. The current *Triticeae* logs quantify the relative cost of read QC/preparation versus clustering, which is useful for scheduling and capacity planning. Caching effectiveness is a key design motivation and is supported by parameter-aware cache boundaries and token-/signature-based reuse logic described in the *Results*.

By producing operational evidence as part of routine execution (structured progress summaries and validation reports), REAPER reduces the gap between “a runnable pipeline” and an auditable analysis process that can be iterated and debugged in long-lived projects.

Recent work in the broader repeat/satellite tooling ecosystem also highlights complementary directions such as tandem-repeat annotation in assemblies and visualization of massive tandem repeat structures (Wlodzimierz et al., 2023; Vollger et al., 2022).

REAPER also has clear limitations. It currently depends on the RepeatExplorer2/TAREAN stack and on access to a Linux-based HPC environment with sufficient memory for graph-based clustering, which may restrict adoption in settings where only web-based Galaxy instances are available. The quality of BLAST-based annotation and comparative summaries is bounded by the completeness and curation of the underlying repeat databases, and careful configuration of read budgets and thresholds remains necessary to avoid misleading abundance estimates. In comparative runs, proportional allocation additionally relies on user-provided genome-size estimates; inaccurate estimates may bias read allocation and downstream abundance comparisons. In particular, REAPER does not yet emit an automated warning when a large disparity in per-sample genome size skews the resulting read-depth ratio enough to risk undersampling low-abundance satellites in the larger genome; adding this check to the existing configuration-validation layer (see *Caching, validation, and monitoring*) is a natural, low-risk extension. Although comparative workflow wiring and freshness-gated post-processing are implemented, quantitative evidence in this report emphasizes solo *Triticeae* rule-level performance logs; comparative runs are documented as illustrative operational snapshots.

Future work includes providing support for user-reported issues on the published tool’s GitHub repository, producing multiple publications on *Triticeae* repeatome evolutionary studies using it, support for additional clustering engines, tighter provenance capture (e.g., configuration and environment snapshots alongside outputs), expanded monitoring for multiproject deployments, and a systematic evaluation of (i) cache effectiveness under common update scenarios and (ii) stability of repeat abundance and classification under controlled configuration changes.

## Data Availability

The datasets presented in this study can be found in online repositories. The names of the repository/repositories and accession number(s) can be found below: https://github.com/Stathmin/REAPER/ https://www.ncbi.nlm.nih.gov/bioproject/PRJNA1494421/.
