# Supplementary material for: REAPER: a project-centric workflow layer for comparative repeatome analysis
Source: Front Plant Sci. 2026 Aug 31;17:1855960. doi: 10.3389/fpls.2026.1855960 (PMC13572731; doi:10.3389/fpls.2026.1855960)
Supplement: Supplementary file 1 [file Table2.docx]

Supplementary Material

# 1 Supplementary File S1. Example of the REAPER global configuration file.

```
global:
  default_threads: 28
  default_memory: 180G
  cache_dir: .snakemake/cache
  log_dir: logs
  temp_dir: tmp
  pythonhashseed: "0"
  total_reads_per_assembly: 8000
  cleanup_after_prepare: true
  incremental_processing: true
  iterative_assembly:
    enabled: false
    depth: 1
    mode: both
  deconseq:
    removeifeitherbad: true
    iterative_reference_coverage_fraction: 0.5
  ncbi_gathering:
    enabled: true
    default_taxonomy_id: "147389"
    max_age_days: 30
projects:
  triticeae_F21FTSEUHT1241:
    taxonomy: Triticeae
    taxonomy_id: "147389"
    total_reads_per_assembly: 50000
    ncbi_repeats: data/ncbi_repeats_triticeae.fa
    oligo_fasta: references/oligo.fasta
    tarean_params:
      threads: 28
      memory_limit: 80%
      r_value: 80%
      mincl: 0.0001
    iterative_assembly:
      enabled: true
      depth: 3
```
